# Genetic polymorphism of the drug-metabolizing enzyme Cytochrome P4502E1 (CYP2E1) in a healthy Saudi population

**DOI:** 10.1016/j.jsps.2021.09.013

**Published:** 2021-10-06

**Authors:** Lenah S. Binmahfouz, Amina M. Bagher

**Affiliations:** Department of Pharmacology and Toxicology, Faculty of Pharmacy, King AbdulAziz University, Jeddah, Saudi Arabia

**Keywords:** *CYP2E1* gene, Polymorphisms, CYP2E1*1B, CYP2E1*5B, CYP2E1*6

## Abstract

**Objectives:**

Cytochrome P450 2E1 (*CYP2E1*) is one of the major enzymes involved in the metabolism and detoxification of various drugs and xenobiotics. Polymorphisms in the *CYP2E1* gene exhibit high inter-individual variations associated with alterations in *CYP2E1* gene expression and enzyme function. This study aimed to determine the genotype distributions and allele frequencies of CYP2E1*1B, *5B, and *6 polymorphisms among Saudis in western Saudi Arabia.

**Methods:**

In total, 140 healthy Saudis attending King Abdulaziz University Hospital between February and April 2021 were included in the study. *CYP2E1* gene polymorphisms were determined by polymerase chain reaction-restriction fragment length polymorphism analysis.

**Results:**

The genotype frequencies of CYP2E1*1B A2A2, A2A1, and A1A1 were 54.29%, 40%, and 8%, respectively. The frequencies of CYP2E1*5B c1c1 and c1c2 genotypes were approximately 99.93% and 0.07%, respectively. The frequencies of the CYP2E1*6 DD, DC, and CC genotypes were 91.43%, 7.85%, and 0.72%, respectively. The genotype distributions for these polymorphisms were consistent with the expected distribution based on Hardy-Weinberg equilibrium. The allele frequencies were 74.29% A2 and 25.71% A1 for CYP2E1*1B, 99.64% c1 and 0.36% c2 for CYP2E1*5B, and 95.36% D and 4.65% C for CYP2E1*6.

**Conclusion:**

The genotype distribution of CYP2E1*1B polymorphism was higher in the western Saudi population, whereas the CYP2E1*5B and *6 polymorphisms were lower than the global average. Knowledge of the prevalence of *CYP2E1* polymorphisms among our population will provide a better understanding of whether individual patients might benefit from their medication or whether they might develop adverse effects.

## 1 Introduction

Cytochrome oxidase P450 (CYP) is one of the major enzymes responsible for the phase I metabolism of various environmental chemicals, carcinogens, and drugs (Guengerich et al., 2016). Based on amino acid sequence homology, CYP is divided into 18 families and 43 subfamilies in humans (Ingelman-Sundberg, 2005, Nelson et al., 2004). Different studies have shown genetic polymorphism with allelic variants occurring in most members of the CYP families, resulting in alterations in the expression or activity of their corresponding enzymes (Waring, 2020). The prevalence of this genetic polymorphism varies significantly among different human populations.

Cytochrome P450 family 2 subfamily E member 1 (*CYP2E1*) has attracted more interest than the other CYPs due to its role in the bioactivation of low-molecular-weight xenobiotics and their transformation into potential carcinogenic or hepatotoxic metabolites (Chen et al., 2019). Genetic polymorphisms in *CYP2E1* have been extensively studied in different populations and have shown high inter-individual variability in their effects on drug metabolism, adverse drug reactions, drug responsiveness, and some of them have been associated with susceptibility to cancer (Soya et al., 2005, Ulusoy et al., 2007, Shahriary et al., 2012, Saeed et al., 2013).

To date, about 100 single nucleotide polymorphisms (SNPs) have been identified for *CYP2E1*. The most common SNPs associated with high inter-individual variations and alterations in *CYP2E1* gene expression and functions include CYP2E1*1B (Brockmöller et al., 1996, McBride et al., 1987), CYP2E1*5B (Hayashi et al., 1991, Watanabe et al., 1990), and CYP2E1*6 (Persson et al., 1993). CYP2E1*1B (dbSNP rs2070676) is a TaqI polymorphism that codes for a base change at 9896C > G, located in intron 7 of *the* CYP2E1 gene. The CYP2E1*1B SNP enhances the activity of the *CYP2E1* enzyme *in vivo* (Haufroid et al., 2002, 2001). CYP2E1*5B contains the polymorphisms RsaI (-1053C > T) (dbSNP rs2031920) and PstI (dbSNP rs3813867) (-1293 G > C) in the 5′ flanking region of the *CYP2E1* gene, and these polymorphisms can alter the transcription rates (Hayashi et al., 1991). CYP2E1*6 (rs6413432), or the DraI polymorphism, is located in intron 6 and is characterized by a 7632 T > A base change. *In vivo,* the CYP2E1*6 polymorphism causes a reduction in *CYP2E1* enzyme activity (Haufroid et al., 2002).

*CYP2E1* metabolizes different prescription drugs, including acetaminophen, isoniazid, chlorzoxazone, and fluorinated anesthetics (Tanaka et al., 2000). For instance, acetaminophen (a commonly used analgesic drug) is safely metabolized in the liver and excreted in the urine. However, a small percentage of acetaminophen is converted by the *CYP2E1* enzyme into a highly toxic byproduct (N-acetyl-p-benzoquinone imine (NAPQI)), which is immediately detoxified in the liver primarily by glutathione conjugation (Gonzalez, 2007, Neafsey et al., 2009). High doses of acetaminophen can lead to acute hepatic failure and, in some cases, death due to high levels of NAPQI and glutathione depletion. Previous studies have reported that CYP2E1*1B polymorphism enhances the activity of the *CYP2E1* enzyme, thereby increasing the metabolism of its substrates to their toxic metabolites (Haufroid et al., 2002, Haufroid et al., 2001, Mazaleuskaya et al., 2015). Moreover, Ueshima et al. (1996) previously reported that the half-life and the elimination rate of acetaminophen were strongly affected by different *CYP2E1* genotypes in alcoholic patients.

The *CYP2E1* enzyme is also involved in the metabolism of isoniazid. This drug is used in combination with pyrazinamide and rifampin to treat tuberculosis infection. Isoniazid is mainly metabolized by N-acetyltransferase 2 (NAT2) and *CYP2E1* enzymes to produce the toxic metabolites acetyl denizens, ketene, and acetylorium ion. A previous study by Yu et al. (2020) reported that CYP2E1*1B polymorphism was associated with an increased risk of isoniazid-induced hepatotoxicity.

The prevalence of *CYP2E1* genetic variation in the western Saudi population has not yet been investigated. Therefore, the aim of the current study was to determine the genotype distributions and allele frequencies of the three most common polymorphisms (CYP2E1*1B, *5B, and *6) among healthy Saudis attending King Abdulaziz University Hospital (KAUH) in Jeddah. This cohort represents the Saudi Arabian population in the western region of the Kingdom of Saudi Arabia.

## 2 Materials and methods

### 2.1 Sample collection

The study was conducted at KAUH in Jeddah, Saudi Arabia, from February to April 2021. The study protocol was approved by the Human Research Ethics Committee of the School of Dentistry at King Abdulaziz University in Jeddah, Saudi Arabia (211–01-21). Saliva samples were collected from 140 unrelated Saudi subjects (70 males and 70 females, age 10**–**60 years old) by a single investigator. All these subjects were attending KAUH for dental check-ups. Inclusion criteria were: 1) Saudi, 2) healthy, and 3) not consuming any prescribed medications. The objective and nature of the study were introduced to the eligible subjects. All participants were required to sign a written consent before enrolment in the study. Genomic DNA was extracted from the saliva samples using the Oragene®-Discover kit (Oragene.One, Canada), following the manufacturer's instructions.

### 2.2 Polymerase chain reaction-restriction fragment length polymorphism (PCR-RFLP)

**Fig. 1:**
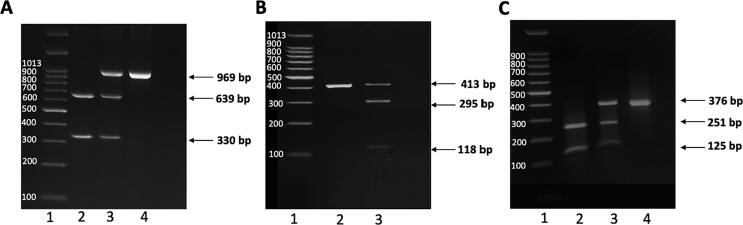
Representative agarose gel images of CYP2E1*1B, CYP2E1*5B and CYP2E1*6 in healthy Saudi samples. (A) Agarose gel (2%) electrophoresis for PCR products of CYP2E1*1B digested with TaqI. Lane 1: 100 bp DNA Molecular Weight Marker, lane 2: A2A2 genotype (639 and 330 bp bands), lane 3: A2A1 genotype (969, 639 and 330 bp bands), and lane 4: A1A1 genotype (969 bp band). (B) Agarose gel (2%) electrophoresis for PCR products of CYP2E1*5B digested with PstI. Lane 1: 100 bp DNA marker, lane 2: c1c1 genotype (413 bp band), and lane 3: c1c2 genotype (413, 295 and 118 bp bands). (C) Agarose gel (2%) electrophoresis for PCR products of CYP2E1*6 digested with DraI: Lane 1: 100 bp DNA marker, lane 2: DD genotype (251 and 125 bp bands), lane 3: DC genotype (376, 251 and 125 bp bands), and lane 4: CC genotype (376 bp band).

PCR-RFLP analysis was utilized to detect *CYP2E1* polymorphism using a previously described protocol (Soya et al., 2005). Briefly, the PCR was performed in a final volume of 20 μl that includes 10 μl of 2X GoTaq® Green Master Mix (Promega), 0.5 μM of both forward and reverse primers (Macrogen), and 100 ng of genomic DNA. The PCR program was as follows: initial denaturation step at 95 °C for 10 min; 30 cycles consisting of denaturation at 95 °C for 1 min, primer-specific annealing temperature for 30 sec, and extension at 72 °C for 1 min; followed by a final extension cycle at 72 °C for 7 min. 10 μl of each PCR product was digested overnight using the specific restriction enzyme (Thermo Scientific) for each polymorphism. Finally, the digested products were fragmented on 2% agarose gel using ENDURO™ Horizontal Electrophoresis Systems (Labnet) at 100 V for 90 mins and visualized using UVP BioDoc-it^TM^ imaging system. The size of each product was determined using a 100 bp DNA ladder. The specific primer pairs, the PCR annealing temperature, and the specific digestive enzymes for each polymorphism are listed in Table 1. The representative agarose gel images of CYP2E1*1B, 5B, and *6 PCR-RFLP assays (Fig. 1) were used to interpret the results of the current study.

**Table 1:** PCR primer sequences, PCR conditions, restriction enzymes and expected PCR products for CYP2E1*5B, CYP2E1*1B, and CYP2E1*6.

SNP	Primer	Annealing Temperature	Enzyme	PCR Product Size (bp)
CYP2E1*1B	F-5′ GGATGATGGGTGGATGCC 3′R-5′ CACATGTGGAGGGGAGAT 3′	58 °C	TaqI	A2A2-639 and 330 bp A2A1-969, 639 and 330 bp A1A1-969 bp
CYP2E1*5B	F-5′ CCAGTCGAGTCTACATTGTCA3′ R-5′ TTCATTCTGTCTTCTAACTGG 3′	55 °C	RsaI/PstI	RsaI c1c1-352 and 61 bp c1c2-413, 352 and 61 bp c2c2-413 PstI c1c1-413 c1c2-413 + 118 + 295 c2c2-118 + 295
CYP2E1*6	F-5′AGTCGACATGTGATGGATCCA 3′ R-5′GACAGGGTTTCATCATGTTGG 3′	64 °C	DraI	DD-251 and 125 bp DC-376, 251 and 125 bp CC-376 bp

### 2.3 Data analysis

The allele and genotype frequencies of *CYP2E1* polymorphisms were calculated by direct counting and compared using a Chi-Square (χ^2^) test with the expected values calculated using the Hardy-Weinberg equilibrium. The frequencies are given for each allele and genotype together with the 95% confidence intervals. The confidence intervals were measured using the normal approximation with continuity correction. Comparisons of allelic frequencies between the Saudis in the Western region and other populations were calculated by the χ^2^ test. *P <* 0.05 was considered statistically significant.

## 3 Results

We examined the genotype distributions for the CYP2E1*1B, *5B, and *6 polymorphisms in 140 participants. The genotype distributions for CYP2E1*1B were as follows: the homozygous wildtype (A2A2) genotype was present in 54.29% (95% C.I. 43.71**–**64.02) of the sample, the heterozygous genotype (A2A1) was present in 40% (95% C.I. 21.26**–**36.81), and the homozygous variant (A1A1) was present in the remaining 8% (95% C.I. 1.59**–**0.90) (Table 2). The allele frequencies of this polymorphism were 74.29% A2 and 25.71% A1 (Table 3).

**Table 2:** Genotype distribution of CYP2E1*1B, *5B, and *6 in 140 Saudi subjects.

SNP	Genotype	Total (n = 140)	Observed Frequency% (95% CI)	Observed/Expected frequencies (under the HW law)	*P* value for HW equilibrium
CYP2E1*1B	A2A2	76	54.29 (43.71 to 64.02)	0.98	0.57
	A2A1	56	40 (21.26 to 36.81)	1.05	
	A1A1	8	5.71 (1.59 to 0.90)	0.86	
CYP2E1*5B	c1c1	139	99.93 (96.08 to 99.98)	1.00	0.97
	c1c2	1	0.07 (0.02 to 3.92)	1.00	
	c2c2	0	0.00 (0)	0.00	
CYP2E1*6	DD	128	91.43 (56.49 to 72.86)	1.41	0.18
	DC	11	7.85 (3.99 to 13.62)	0.88	
	CC	1	0.72 (0.02 to 3.92)	1.05	

SNP: Single nucleotide polymorphism. CI: Confidence interval. HW: Hardy-Weinberg.

**Table 3:** CYP2E1*1B, *5B, and *6 allele frequency in the Saudi population.

SNP	Allele	Frequency % (95% CI)
CYP2E1*1B	A2	74.29 (66.22 to 81.29)
	A1	25.71 (18.71 to 33.78)
CYP2E1*5B	c1	99.64 (96.08 to 99.98)
	c2	0.36 (0.02 to 3.92)
CYP2E1*6	D	95.36 (90.91 to 98.41)
	C	4.65 (2.03 to 10.03)

The genotype frequencies of CYP2E1*5B were as follows: the homozygous wildtype (c1c1) was present in 99.93% (95% C.I. 96.08**–**99.98) of the sample, the heterozygous genotype (c1c2) was present in 0.07% (C.I. 0.02**–**3.92), and the homozygous variant (c2c2) genotype was not detected in this population (Table 2). The allele frequencies of CYP2E1*5B were 99.64% c1 and 0.36% c2 (Table 3).

The genotype frequencies of CYP2E1*6 were as follows: the homozygous wildtype (DD) genotype was present in 91.43% (95% C.I. 56.49**–**72.86) of the sample, the heterozygous genotype (DC) was present in 7.85% (95% C.I. 3.99**–**13.62), and the homozygous variant (CC) was present in only 0.72% (95% C.I. 0.02**–**3.92). The allele frequencies of CYP2E1*6 polymorphism were 95.36% and 4.65% for D and C, respectively (Table 3).

All the genotype distributions for CYP2E1*1B, CYP2E1*5B, and CYP2E*6 polymorphisms were consistent with the Hardy-Weinberg equilibrium (*P*-value > 0.05, Table 2).

## 4 Discussion

The polymorphisms CYP2E1*1B, *5B, and *6 have received much attention because of their association with inter-individual variation, altered gene expression, and modified enzyme function. The aim of the current study was to determine the genotype distribution and allele frequencies of CYP2E1*1B, *5B, and *6 polymorphisms among healthy Saudis in Jeddah, Kingdom of Saudi Arabia.

The total population of Saudi Arabia is 90% Arab and 10% Afro-Asian (Saudi Arabia, xxxx). The presence of the two holy cities of Islam (Makkah and Madinah) in the western region of Saudi Arabia has led many Muslims to settle there. With an estimated population of 3.976 million, Jeddah is the second-most populous city in the western region of Saudi Arabia (*Saudi Arabia—The World Factbook*). However, few pharmacogenomics studies have examined the influence of genetic variation in this population with respect to their response to various drugs. The current study is the first to report the prevalence of CYP2E1*1B, *5B, and *6 polymorphisms in the western Saudi population. We found that CYP2E1*1B (rs2070676) polymorphism was present in 45.71% of our sample. The CYP2E1*5B RsaI (rs2031920) and *PstI* (drs3813867) polymorphisms were present in only 0.07% of the sample, while the CYP2E1*6 (rs6413432) polymorphism was present in 8.57% of the tested samples. CYP2E1*1B polymorphism was more common than CYP2E1*6 polymorphism*.* By contrast, CYP2E1*5B polymorphisms were not identified in the population under study.

Comparison of CYP2E1*1B polymorphism in the current study (45.71%) to the global average (30.73%, dbSNP rs2070676) reveals a higher frequency of CYP2E1*1B polymorphism in the Jeddah population. As shown in Table 4, the CYP2E1*1B A2A1 and A1A1 genotypes were present in 40.0% and 5.72% of the current sample, respectively, and these proportions are significantly higher than those reported for the Caucasian population (24% and 1%, respectively, *P* < 0.001). Similarly, previous studies have reported a high CYP2E1*1B frequency in Asians (18.1%) and an even higher frequency in Africans (65.9%) (Lee et al., 2008).

**Table 4:** Comparison of *CYP2E1* genotype polymorpshim in the Western region of Saudi Arabia compared to other populations.

Genotype	Saudis (Western Region)		Saudis (Central Region)		Turkish		Iranians		South Indian (Tamilians)		Caucasians	
	n	%	n	%	n	%	n	%	n	%	n	%
CYP2E1*1B	140		NR		NR		NR		123 (NS)		375 (*P* < 0.001)	
A2A2	76	54.29	–		–		–		75	61	279	75
A2A1	56	40							44	35.8	91	24
A1A1	8	5.71							4	3.2	5	1
CYP2E1*5B	140		NR		206 (NS)		200 (NS)		123 (NS)		375 (*P* = 0.006)	
c1c1	139	99.93	–		198	96.12	194	97	122	99.2	350	93
c1c2	1	0.07			8	3.88	6	3	1	0.80	25	7
c2c2	0	0			0	0	0	0	0	0	0	0
CYP2E1*6	140		79 (*P* < 0.001)		206 (NS)		200 (*P* < 0.001)		123 (*P* = 0.001)		375 (*P* = 0.015)	
DD	128	91.43	51	64.55	173	83.98	138	69	88	71.5	305	81
DC	11	7.85	28	35.44	32	15.53	60	30	31	25.25	68	18
CC	1	0.72	0	0	1	0.49	2	1	4	3.25	2	1
Reference	Present study		Saeed et al., 2013		Ulusoy et al., 2007		Shahriary et al., 2012		Soya et al., 2005		Wong et al., 2000	

n: Number of subjects. NR: Not reported. NS: Not significant. P values were calculated using the Chi square test and indicate significant difference between the Saudis in the Western region and other populations.

The genotype distribution of the CYP2E1*5B polymorphism observed in the western Saudi population is consistent with the distribution in other populations across the world, according to the dbSNP database (rs1801131). The CYP2E1*5B RsaI (dbSNP rs2031920) and *PstI* (dbSNP rs3813867) polymorphisms were present in only 0.07% of the sample, compared to the global averages of 6.00% and 8.5%, respectively (rs1801131). The homozygous (c1c1) genotype frequency in the current study is 99.9%, which is comparable to the frequency in other populations: 96.1% in a Turkish sample (Ulusoy et al., 2007), 97% in Iranians (Shahriary et al., 2012), and 99.2% in Tamils (Soya et al., 2005), with no significant differences (Table 4, *P* > 0.05). Similarly, the heterozygous (c1c2) genotype frequency is lower in western Saudis (0.07%), compared to Turkish (3.8%), Iranian (3%), and Tamil (0.8%) populations. When compared to Caucasians, the c1c2 genotype occurred at a significantly lower frequency in Jeddah (Wong et al., 2000; P = 0.006). Interestingly, the mutant c2c2 was absent in all populations in this comparison, confirming its rare occurrence. This indicates that any influence of CYP2E1*5B on drug metabolism is likely to be minimal in our population.

The frequencies of CYP2E1*6 polymorphism show the greatest inter-ethnic variation. In Saudi Arabia, a previous study reported a prevalence of 35.44% for the CYP2E1*6 polymorphism in healthy subjects from the central region of Riyadh (Saeed et al., 2013). By comparison, a significant difference was found between the prevalence of the CYP2E1*6 polymorphism in the central and western regions (Table 4; *P* < 0.001). The heterozygous DC genotype occurred with a frequency of 7.85% in the current study, which is significantly lower than the 35.44% reported in Riyadh. This difference could be attributed to geographical locations, genetic backgrounds, lifestyle factors, and dietary habits, affecting *CYP2E1* polymorphism. The mutant (CC) allele was not identified in any of the 79 subjects studied in the central region. However, this was only the control (healthy) group in the study by Saeed et al. (2013), so the sample size was relatively small for pharmacogenetics research.

A significant difference was observed in frequency percentages for DC and CC of CYP2E1*6 in this population compared with the values reported for Iranians, South Indians, and Caucasians. Still, they did not differ from the reported values for the Turkish population (Table 4). The mutant CC genotype occurred with a frequency of 0.72% in the current study, and this value was significantly lower than the frequency reported for Iranians (1%), South Indians (3.25%), and Caucasians (1%) (Table 4). The CYP2E1*6 polymorphism was present in only 8.57% of the tested samples, far lower than the global average (16.07%) reported in the dbSNP database (rs6413432).

The *CYP2E1* enzyme metabolizes xenobiotics into potential carcinogenic or hepatotoxic metabolites (Chen et al., 2019). In the current study, we observed a higher frequency of CYP2E1*1B polymorphism in the healthy western Saudi population compared to the global average. The high frequency of this polymorphism may contribute to inter-individual variability in drug metabolism and the development of adverse drug reactions for prescribed drugs such as acetaminophen and isoniazid. Acetaminophen is highly used as an over-the-counter analgesic in Saudi Arabia (Babakor, 2018). The CYP2E1*1B genotype has been reported to increase the conversion of acetaminophen to NAPQI, thereby increasing the risk of hepatotoxicity (Gonzalez, 2007, Neafsey et al., 2009). Given the higher prevalence of CYP2E1*1B polymorphism among the Western Saudi population, an increase in the incidence of acetaminophen-induced adverse drug reactions including hepatotoxicity following the administration of overdoses or long-term clinical use of therapeutic doses of acetaminophen might be observed.

A previous study by Yu et al. (2020) reported that CYP2E1*1B polymorphism was associated with an increased risk of isoniazid-induced hepatotoxicity. Furthermore, a recent study by Zahra et al. (2020) revealed a high frequency of the NAT2 gene with slow acetylators in the Saudi population. Importantly, the combination of the CYP2E1*1B genotype with a slow acetylator NAT2 genotype increased the risk of isoniazid-induced hepatotoxicity (An et al., 2012). Therefore, Saudis are at higher risk of developing isoniazid-induced hepatotoxicity. In our study, a high frequency of the CYP2E1*1B polymorphism was detected among healthy Saudi, which emphasizes that detailed evaluation and investigation of the patients’ previous medical and family history can be suggested as a valuable tool to diagnose CYP2E1*1B polymorphisms in patients who develop adverse drug reactions following acetaminophen or isoniazid treatment.

In conclusion, the current study provides novel information regarding *CYP2E1* genotype distributions in Jeddah, Saudi Arabia. Future studies with a larger sample size from Saudis across different regions of Saudi Arabia are recommended to obtain an accurate estimation of the prevalence of *CYP2E1* polymorphisms among Saudis. The data on the prevalence of these polymorphisms will assist in optimizing drug responses and in minimizing potential adverse effects. Furthermore, this knowledge will help in predicting susceptibility to various cancers and liver diseases.

## Declaration of Competing Interest

The authors declare that they have no known competing financial interests or personal relationships that could have appeared to influence the work reported in this paper.
